# Losartan ameliorates TGF-**β**1–induced CFTR dysfunction and improves correction by cystic fibrosis modulator therapies

**DOI:** 10.1172/JCI155241

**Published:** 2022-06-01

**Authors:** Michael D. Kim, Charles D. Bengtson, Makoto Yoshida, Asef J. Niloy, John S. Dennis, Nathalie Baumlin, Matthias Salathe

**Affiliations:** Department of Internal Medicine, Division of Pulmonary, Critical Care and Sleep Medicine, University of Kansas Medical Center, Kansas City, Kansas, USA.

**Keywords:** Inflammation, Pulmonology, Ion channels

## Abstract

Highly effective modulator therapies dramatically improve the prognosis for those with cystic fibrosis (CF). The triple combination of elexacaftor, tezacaftor, and ivacaftor (ETI) benefits many, but not all, of those with the most common F508del mutation in the CF transmembrane conductance regulator (CFTR). Here, we showed that poor sweat chloride concentration responses and lung function improvements upon initiation of ETI were associated with elevated levels of active TGF-**β**1 in the upper airway. Furthermore, TGF-**β**1 impaired the function of ETI-corrected F508del-CFTR, thereby increasing airway surface liquid (ASL) absorption rates and inducing mucus hyperconcentration in primary CF bronchial epithelial cells in vitro. TGF-**β**1 not only decreased *CFTR* mRNA, but was also associated with increases in the mRNA expression of *TNFA* and *COX2* and TNF-**α** protein. Losartan improved TGF-**β**1–mediated inhibition of ETI-corrected F508del-CFTR function and reduced *TNFA* and *COX2* mRNA and TNF-**α** protein expression. This likely occurred by improving correction of mutant CFTR rather than increasing its mRNA (without an effect on potentiation), thereby reversing the negative effects of TGF-**β**1 and improving ASL hydration in the CF airway epithelium in vitro. Importantly, these effects were independent of type 1 angiotensin II receptor inhibition.

## Graphical abstract

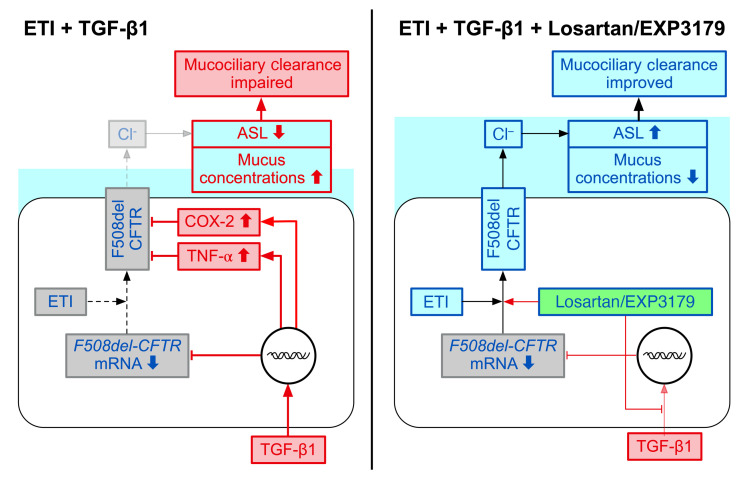

## Introduction

Recent advances in small molecule therapies have greatly improved the outlook for those with cystic fibrosis (CF) caused by certain mutations in the CF transmembrane conductance regulator (*CFTR*). These highly effective modulator therapies target the underlying defects in the CFTR protein (1). The latest combination of elexacaftor, tezacaftor, and ivacaftor (ETI or TRIKAFTA) combines 2 correctors (ET) and a potentiator (I), producing remarkable improvements in lung function in many, but not all, CF patients carrying at least one copy of the most common CFTR mutation, F508del (2–4). Heterogeneous responses to ETI (4) suggest that additional factors can influence the efficacy of even highly effective CFTR modulators.

The efficacy of modulator therapy in a CF inflammatory airway environment has been controversial, with some studies showing improved functional CFTR recovery (5, 6). However, the cytokine TGF-β1 is not only linked to worse pulmonary outcomes in CF (7, 8), but can also diminish the efficacy of first/second generation modulators to rescue mutant CFTR function in vitro (9) by reducing the expression of *CFTR* mRNA (9–11). TGF-β1 can also induce the expression of other proinflammatory mediators that influence the efficacy of CFTR modulators: an example is COX2 (12) and its major enzymatic product, prostaglandin E2 (PGE2), which are both elevated and themselves considered mediators of inflammation (13). Not only are both COX2 and PGE2 implicated in CF lung disease (14, 15), but the ability of ibuprofen, a nonselective COX inhibitor, to slow lung disease progression in pediatric CF patients (16) points to an important role for COX2 in the pathophysiology of CF pulmonary disease.

Losartan, used clinically as a type 1 angiotensin II receptor blocker (ARB) for treatment of hypertension, is known to inhibit TGF-β signaling and exert antiinflammatory properties independently of its receptor-blocking action. We previously demonstrated that losartan and its anti–TGF-β metabolite EXP3179, which has no angiotensin receptor–blocking properties, could reverse TGF-β1–induced mucociliary dysfunction in an ovine model of CF-like airway disease in vivo and in CF bronchial epithelial (CFBE) cells in the absence of highly effective CFTR modulators in vitro (17). This is achieved, in part, by the ability of losartan to reverse TGF-β1–induced dysfunction of the large conductance, Ca^2+^-activated, and voltage-dependent K^+^ (BK) channels that are important for airway surface liquid (ASL) hydration (17–20). In this study, we demonstrate that TGF-β1 reduces the efficacy of ETI on functional F508del-CFTR recovery, causing impairments in mucociliary clearance in primary CFBE cells in vitro. Losartan reversed TGF-β1–induced mucociliary dysfunction through a corrector mechanism that likely involves reducing TNF-α and COX2 expression. Importantly, those with CF who had worse lung function responses to ETI had higher levels of TGF-β1 activity in the upper airway, demonstrating the clinical relevance of these studies.

## Results and Discussion

### Expression of TGF-β1 is associated with worse response to ETI in CF patients.

**Figure 1:**
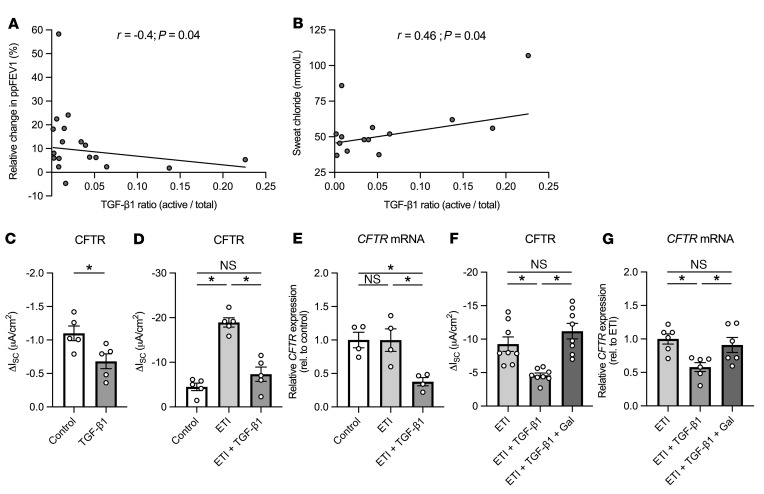
Effects of TGF-β1 on ETI efficacy in CF patients in vivo and homozygous F508del CFBE cells in vitro. (**A** and **B**) TGF-β1 correlations with outcome parameters after starting CF patients on ETI. (**A**) TGF-β1 (ratio of active/total) in nasal fluid collected with Leukosorb from CF patients on ETI is inversely correlated with ppFEV1 improvement within 3 months of starting ETI. TGF-β1 was measured both in its active and total form and is expressed as a ratio. (**B**) TGF-β1 is positively correlated with sweat chloride values (higher values indicate less CFTR activity, which shows worse response to ETI). Statistics (**A** and **B**): Spearman’s correlation coefficient, 1-tailed *P* values. (**C** and **D**) Basolateral TGF-β1 (5 ng/mL) causes a significant decrease in noncorrected (**C**) and ETI-corrected (**D**) F508del-CFTR conductance after 24 hours. *n* = 5, ≥2 CF lungs. I_SC_, short circuit current. (**E**) *CFTR* mRNA expression levels are significantly reduced in ETI-treated CFBE cells 24 hours after basolateral exposure to TGF-β1. *n* = 4, 4 CF lungs. (**F** and **G**) Galunisertib (Gal) (10 μM) rescues the effects of TGF-β1 on ETI-corrected F508del-CFTR function (**F**) and *CFTR* mRNA expression (**G**). *n* = 8, 3 CF lungs. Statistics (**C**–**G**): Data are shown as mean ± SEM. *P < 0.05. Student’s *t* test (**C**) and 1-way ANOVA followed by Holm-Šidák (**D**–**G**) after assessing normality by Shapiro-Wilk.

To determine whether the in vivo response to ETI is affected by airway TGF-β1 activity, we collected nasal epithelial lining fluid from CF participants with at least 1 copy of F508del who were on ETI at the time (Table 1). Nasal mucosal samples were used because they are an accessible surrogate for lower airways (21). We determined associations between TGF-β1 activity and sweat chloride concentration (an indirect measure of CFTR activity) as well as change in lung function in CF participants after starting ETI (<3 months). There was a significant correlation between levels of active TGF-β1 (expressed as a ratio of active/total TGF-β1) and poor improvement in percentage predicted forced expiratory volume in 1 second (ppFEV1) after starting ETI (Figure 1A). There was also a significant correlation between active TGF-β1 and sweat chloride concentrations (Figure 1B). These data suggest that TGF-β1–dominant inflammation reduces ETI efficacy.

### TGF-β1 inhibits ETI-corrected F508del-CFTR function in CF air-liquid interface cultures.

We investigated whether TGF-β1 inhibits the correction of F508del-CFTR by ETI in primary CFBE cells homozygous for F508del-CFTR cultured at the air-liquid interface (ALI). Characteristics of CF donor lungs are listed in Supplemental Table 1 (supplemental material available online with this article; https://doi.org/10.1172/JCI155241DS1). Exposure of CFBE cells to recombinant TGF-β1 (5 ng/mL) in the basolateral media significantly reduced both noncorrected and ETI-corrected F508del-CFTR function after 24 hours (Figure 1, C and D). TGF-β1 was previously shown to decrease *CFTR* mRNA levels (9–11). Similarly, we found that 24-hour TGF-β1 exposure decreased expression of *F508del-CFTR* mRNA in CFBE cells treated with ETI (Figure 1E). Importantly, the effects of TGF-β1 on ETI-corrected F508del function and expression were blocked by the TGF-β receptor 1 inhibitor galunisertib, demonstrating a specific action of TGF-β1 (Figure 1, F and G).

### Losartan partially restores F508del-CFTR correction by ETI in TGF-β1–exposed CF ALI cultures.

**Figure 2:**
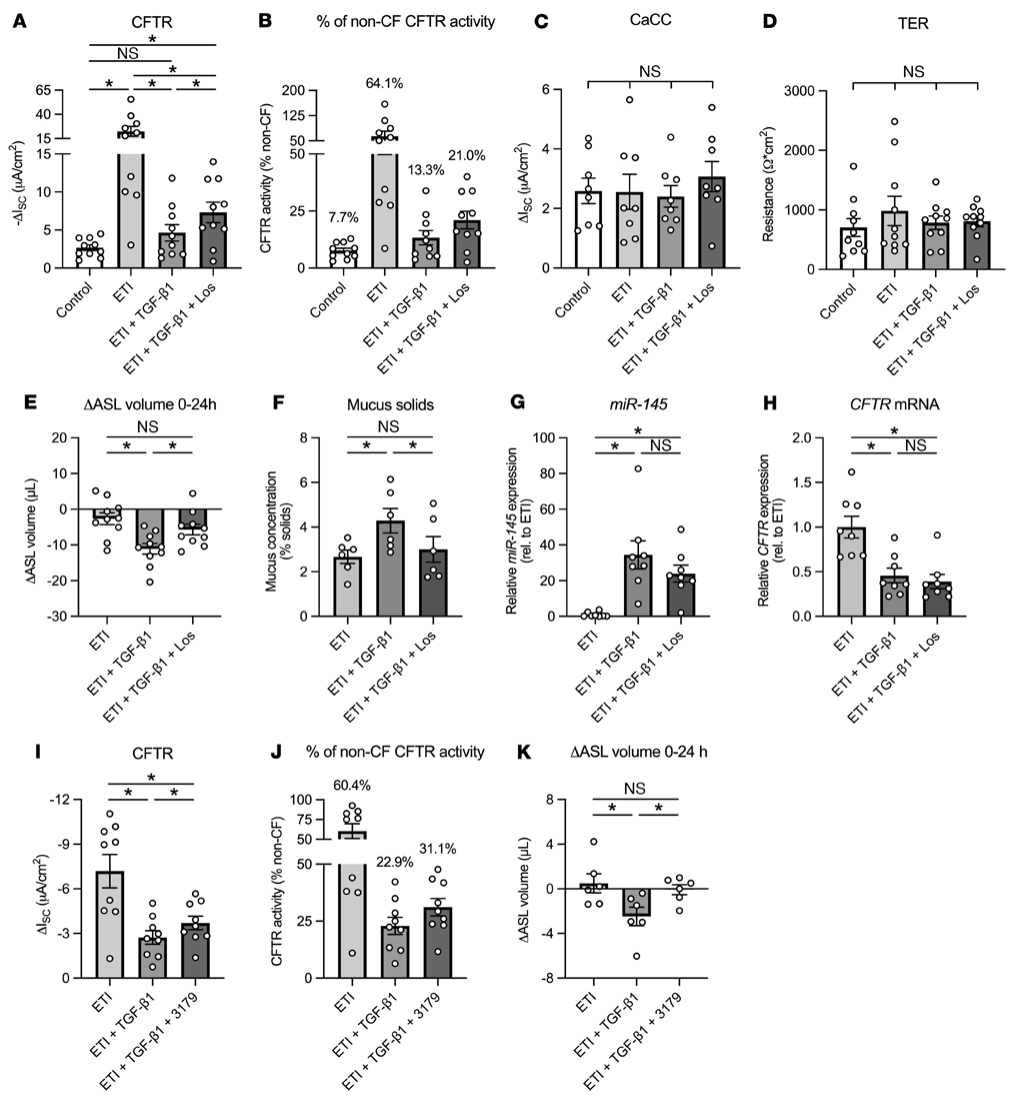
Losartan partially rescues TGF-β1–mediated impairments to ETI-corrected F508del-CFTR function in homozygous F508del CFBE cells in vitro. (**A**) Fully differentiated CFBE cells were exposed to DMSO (Control), ETI, ETI plus TGF-β1 (5 ng/mL), or ETI plus TGF-β1 plus losartan (10 μM). All exposures are 24 hours except for losartan (≥21 days). The TGF-β1–mediated decrease in ETI-corrected F508del-CFTR conductance was partially rescued by losartan. *n* = 10, 5 CF lungs. (**B**) F508del-CFTR currents in **A** are shown as percentages of WT (non-CF) CFTR activity. (**C** and **D**) CaCC conductance (**C**) and transepithelial resistance (TER) (**D**) in ETI-treated F508del CFBE cells were not significantly changed by TGF-β1 in the presence or absence of losartan. *n* ≥ 8, 4 CF lungs. (**E**) Basolateral TGF-β1 induces greater ASL absorption (indicated by a more negative ΔASL volume) in ETI-treated CFBE cells after 24 hours, which is reversed by losartan. *n* = 10, 4 CF lungs. (**F**) TGF-β1 exposure increases mucus concentration (indicated by an increase in % mucus solids) in ETI-treated CFBE cells after 48 hours, which is reversed by losartan. *n* = 6, 3 CF lungs. (**G**) Basolateral TGF-β1 induces a significant increase in the expression of *miR-145* in ETI-treated CFBE cells after 24 hours. Losartan does not reverse the increase in *miR-145* expression. *n* = 8, 5 CF lungs. (**H**) *CFTR* mRNA expression is significantly reduced in ETI-treated CFBE cells 24 hours after TGF-β1 exposure. Losartan does not restore levels of *CFTR* mRNA expression. *n* = 8, 5 CF lungs. (**I**) The TGF-β1–mediated decrease in ETI-corrected F508del-CFTR conductance is partially rescued by EXP3179 (5 μM). CFBE cells were pretreated with EXP3179 for 1 hour before addition of TGF-β1. *n* = 9, 5 CF lungs. (**J**) F508del-CFTR currents in **I** are shown as percentages of WT (non-CF) CFTR activity. (**K**) ASL absorption induced by TGF-β1 in ETI-treated CFBE cells is reversed by EXP3179. *n* = 6, 4 CF lungs. Data are shown as mean ± SEM. *P < 0.05, 1-way ANOVA followed by Holm-Šidák (**A**, **C**, **E**, **F**, **I**, and **K**) and Friedman test (**D**, **G**, and **H**) after assessing normality by Shapiro-Wilk.

Next, we tested to determine whether losartan could ameliorate the effects of TGF-β1 on ETI-corrected F508del-CFTR function in vitro. CFBE cells were treated with losartan (10 μM) in the basolateral media for at least 21 days to allow for accumulation of EXP3179 (17) before the addition of ETI and TGF-β1 (5 ng/mL). Chronic losartan treatment had no impact on the expression of the TGF-β receptor or transepithelial resistance and CFTR activity in CF ALI cultures (Supplemental Figure 1). However, losartan improved F508del-CFTR conductance by a mean of 58% in ETI- and TGF-β1–exposed CFBE cells (Figure 2A). Furthermore, ETI-corrected F508del-CFTR function was restored to 21% of WT CFTR activity with losartan compared with 13.3% without (Figure 2B). CFTR correction that improves activity to more than 10% of the WT level in vitro is considered clinically relevant. In fact, the correction level of CFTR is significantly correlated with FEV1 changes in those with CF on modulators (22–24). Losartan also improved F508del-CFTR conductance in TGF-β1–exposed CFBE cells in the presence of the first-generation CFTR corrector lumacaftor (Supplemental Figure 2). TGF-β1 caused a small but significant reduction in CFTR conductance in ivacaftor-treated G551D/F508del CFBE cells, an effect that was not reversed by losartan (Supplemental Figure 3). TGF-β1 did not affect the conductance of calcium-activated chloride currents (CaCC) or transepithelial resistance of ETI-treated CFBE cells (Figure 2, C and D).

### Losartan rescues TGF-β1–induced ASL absorption and mucus hyperconcentration in ETI-treated CF ALI cultures.

TGF-β1–mediated reduction in F508del-CFTR activity correlated with a significant increase in ASL absorption in CFBE cells after 24 hours despite the presence of ETI (Figure 2E). Losartan reversed TGF-β1–mediated ASL dehydration and restored ASL absorption rates to that of CFBE cells treated with ETI alone (Figure 2E). Losartan similarly improved ASL volumes in TGF-β1– and lumacaftor-treated F508del CFBE cells, but not in TGF-β1– and ivacaftor-treated G551D/F508del CFBE cells (Supplemental Figures 2 and 3). TGF-β1–induced ASL dehydration led to mucus hyperconcentration, as determined by the percentage of mucus solids, in ETI-treated CFBE cells (Figure 2F). Losartan significantly reduced the percentage of mucus solids in TGF-β1–exposed, ETI-treated CFBE cells (Figure 2F).

TGF-β1 has a detrimental impact on ion channels other than CFTR that are important for ASL hydration (17, 19). We found that basolateral TGF-β1 (5 and 10 ng/mL) also significantly reduced BK channel function in CFBE cells homozygous for F508del-CFTR in the presence of tezacaftor and ivacaftor (Supplemental Figure 4). Losartan rescued TGF-β1 inhibition of BK channel activity (Supplemental Figure 4). Thus, it is possible that these ion channels contribute to losartan’s reversal of TGF-β1–induced ASL dehydration.

### Reversal of TGF-β1 inhibition of ETI-corrected F508del CFTR function by losartan seems independent of miR-145 and its ARB ability.

We further investigated the mechanism by which losartan blocks TGF-β1 signaling to improve ETI-corrected F508del-CFTR function. TGF-β1 can induce microRNA-145 (*miR-145*) expression, which downregulates expression of *CFTR* mRNA (9, 25, 26). Basolateral TGF-β1 significantly increased *miR-145* in ivacaftor-treated CFBE cells after 24 hours (Figure 2G). However, losartan did not ameliorate this TGF-β1 effect (Figure 2G). Furthermore, losartan failed to reverse TGF-β1–mediated downregulation of *CFTR* mRNA expression (Figure 2H), suggesting the anti–TGF-β1 action of losartan is unlikely due to regulation of *miR-145*.

EXP3179 (5 μM), with no ARB property, rescued TGF-β1 inhibition of ETI-corrected F508del-CFTR function and restored ASL volumes after 24 hours similarly to losartan, supporting the notion that losartan’s effects do not involve angiotensin receptor blockade (Figure 2, I–K).

### Losartan reduces TGF-β1–induced increases in TNF-α in ETI-treated CF ALI cultures.

**Figure 3:**
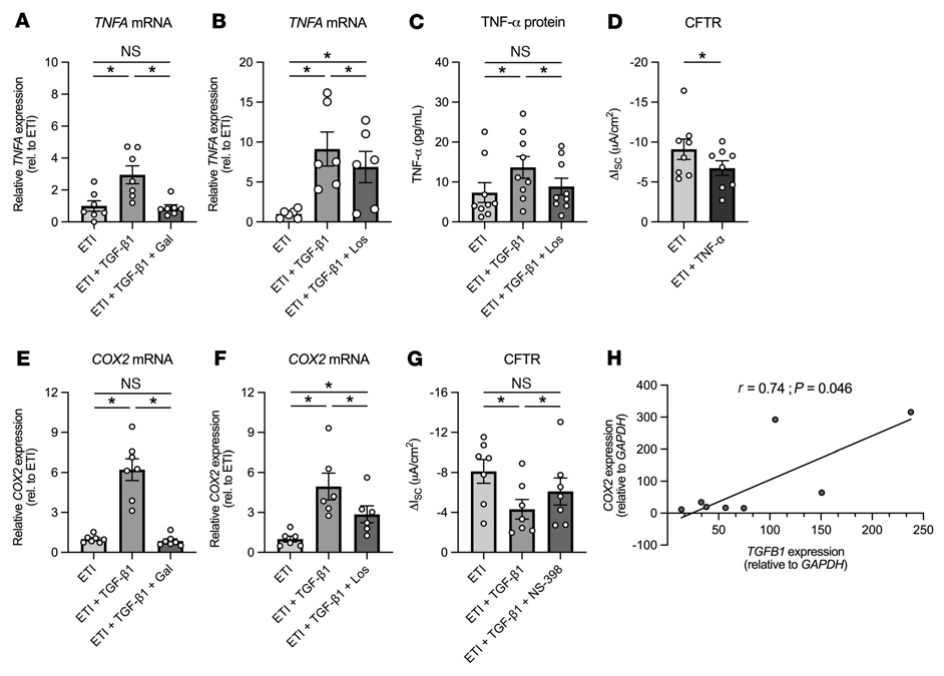
Losartan reverses TGF-β1–induced increases in TNF-α and COX2 expression in ETI-treated homozygous F508del CFBE cells in vitro. (**A**) Galunisertib (10 μM) rescues TGF-β1–induced increases in *TNFA* mRNA expression levels in ETI-treated CFBE cells after 24 hours. *n* = 7, 3 CF lungs. (**B**) Losartan partially inhibits TGF-β1–induced increases in *TNFA* mRNA expression levels after 24 hours. *n* = 6, 3 CF lungs. (**C**) The increase in secreted TNF-α protein in the basolateral media after TGF-β1 exposure is reversed by losartan. *n* = 9, 4 CF lungs. (**D**) Basolateral TNF-β (10 ng/mL) causes a significant reduction in ETI-corrected F508del-CFTR activity. *n* = 8, 4 CF lungs. (**E**) Galunisertib (10 μM) rescues TGF-β1–induced increases in *COX2* mRNA expression levels in ETI-treated CFBE cells after 24 hours. *n* = 7, 3 CF lungs. (**F**) Losartan also reduces TGF-β1–induced increases in *COX2* mRNA expression. *n* = 6, 3 CF lungs. (**G**) The decrease in ETI-corrected F508del-CFTR conductance caused by TGF-β1 exposure is reversed by the COX2 inhibitor NS-398. *n* = 7, 3 CF lungs. (**H**) Correlation between nasal cell *TGFB1* and *COX2* mRNA expression in CF participants on ETI. Only cDNA samples with Ct values below 30 for *GAPDH* were used for analysis. For **A**–**G**, data are shown as mean ± SEM. *P < 0.05, Friedman test (**A**, **C**, and **E**); 1-way ANOVA followed by Holm-Šidák (**B**, **F**, and **G**); and Student’s *t* test (**D**) after assessing normality by Shapiro-Wilk; Spearman’s correlation coefficient, 2-tailed *P* values (**H**).

Compared with ETI treatment alone, exposure of ETI-treated CFBE cells to basolateral TGF-β1 led to significantly increased expression levels of *TNFA* mRNA after 24 hours, and both galunisertib and losartan significantly reduced these effects (Figure 3, A and B). Increased *TNFA* mRNA expression correlated with increased levels of secreted TNF-α protein in basolateral media, which was reversed by losartan (Figure 3C). The role of TNF-α in assisting modulator correction of mutant CFTR function remains unclear (5). Although the combination of TNF-α (10 ng/mL) with IL-17 (20 ng/mL) was recently shown to improve ETI-corrected F508del-CFTR function (5), we found that TNF-α alone (10 ng/mL) reduced the efficacy of ETI in modulating F508del-CFTR function (Figure 3D). TNF-α also induced a significant increase in mRNA expression levels of *COX2* and known downstream cytokines, including *IL1B*, *IL6*, and *IL8* (Supplemental Figure 5). These data suggest that reducing TNF-α may partially explain the ability of losartan to rescue TGF-β1–mediated impairments in ETI-corrected F508del-CFTR function.

### COX2 inhibition partially restores F508del-CFTR correction by ETI in TGF-β1–exposed CF ALI cultures.

We previously found that TGF-β1, in the absence of CFTR modulators, induces *COX2* mRNA expression in CFBE cells (17). *COX2* mRNA is similarly increased in ETI-treated CFBE cells after 24-hour basolateral TGF-β1 exposure, an effect blocked by galunisertib (Figure 3E). Losartan also partially reversed TGF-β1–induced increases in *COX2* mRNA expression (Figure 3F), suggesting that reducing COX2 activity might be an important mechanism of restoring CFTR function. To determine whether COX2 contributes to TGF-β1 inhibition of ETI-mediated F508del-CFTR correction, a selective inhibitor was used (27). NS-398 (10 μM) significantly improved F508del-CFTR activity in TGF-β1–treated CFBE cells in the presence of ETI (Figure 3G). NS-398 alone did not alter CFTR function (Supplemental Figure 6). Finally, we found a significant correlation between *TGFB1* and *COX2* mRNA expression from nasal cells of CF participants on ETI (Figure 3H), suggesting that elevated *COX2* might contribute to the reduced efficacy of ETI in these individuals.

In summary, we show that elevated levels of TGF-β1 in the upper airway correlate with higher sweat chloride concentrations and smaller improvements in ppFEV1 after starting ETI in CF patients. Due to the cross-sectional study design, we were unable to determine whether TGF-β1 levels improved upon initiation of ETI. The small number of participants was also a limitation. Although our in vivo data seemingly contradict recent evidence that some cytokines can improve the efficacy of CFTR modulators (5, 6), airway inflammation varies in its composition, thereby affecting treatment with highly effective modulators differently. In further support of a detrimental role for TGF-β1 in CF, our in vitro data demonstrate that TGF-β1 impairs the function of ETI-corrected F508del-CFTR by reducing *CFTR* mRNA and by increasing expression of *TNFA* and *COX2* mRNA and TNF-α protein.

Losartan and its metabolite EXP3179 can ameliorate the effects of TGF-β1 in ETI-corrected F508del CFBE cells in vitro. This is achieved independently of *CFTR* mRNA regulation. Furthermore, losartan did not rescue TGF-β1 inhibition of CFTR function in ivacaftor-treated G551D/F508del CFBE cells, possibly indicating it works on correction, not potentiation, even though these results were based on a single donor. However, the ability of losartan to reduce *COX2* expression supports the idea that losartan may function in part by abrogating the effects of TGF-β1 on mutant CFTR correction: ibuprofen can itself function as a CFTR corrector via COX inhibition (28). Even though this was reported as COX1 inhibition, these studies were not performed under inflammatory conditions. Furthermore, our data show that blocking COX2 reverses TGF-β1 inhibition of ETI-corrected F508del-CFTR function, providing further evidence that increased *COX2* is harmful in CF pulmonary disease. Finally, losartan reversed TGF-β1–induced increases in TNF-α, which by itself increases *COX2* expression and impairs ETI-corrected F508del function. These studies set the stage for clinical studies using losartan for those with CF who are treated with ETI but show poor clinical responses.

## Methods

Detailed methods are described in Supplemental Methods.

### Study approval.

The study protocol was approved by the University of Kansas Medical Center Institutional Review Board, and informed consent was obtained from each participant.

## Author contributions

MDK, CDB, NB, and MS conceived and designed the study. All authors executed experiments and analyzed the data. CDB, MDK, and MS took part in clinical study recruitment and collection and interpretation of data. MDK, CDB, and MS wrote the manuscript. All authors discussed the results and commented on the manuscript.

## Supplementary Material

Supplemental data

## Figures and Tables

**Table 1:**
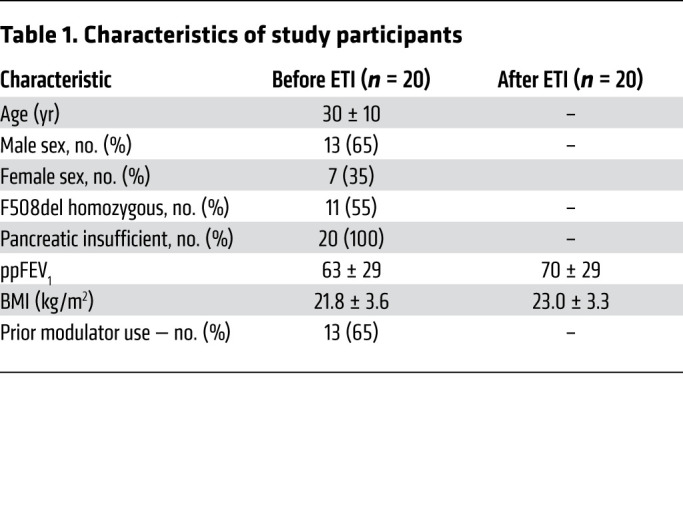
Characteristics of study participants

## Version 1 04/21/2022

In-Press Preview

## Version 2 06/01/2022

Electronic publication

## References

[1] Rowntree RK, Harris A (2003). The phenotypic consequences of CFTR mutations. Ann Hum Genet.

[2] Middleton PG (2019). Elexacaftor-tezacaftor-ivacaftor for cystic fibrosis with a single Phe508del allele. N Engl J Med.

[3] Heijerman HGM (2019). Efficacy and safety of the elexacaftor plus tezacaftor plus ivacaftor combination regimen in people with cystic fibrosis homozygous for the F508del mutation: a double-blind, randomised, phase 3 trial. Lancet.

[4] Keating D (2018). VX-445-tezacaftor-ivacaftor in patients with cystic fibrosis and one or two Phe508del alleles. N Engl J Med.

[5] Rehman T (2021). Inflammatory cytokines TNFα and IL-17 enhance the efficacy of cystic fibrosis transmembrane conductance regulator modulators. J Clin Invest.

[6] Gentzsch M (2021). Airway epithelial inflammation in vitro augments the rescue of mutant CFTR by current CFTR modulator therapies. Front Pharmacol.

[7] Harris WT (2009). Transforming growth factor-beta(1) in bronchoalveolar lavage fluid from children with cystic fibrosis. Pediatr Pulmonol.

[8] Thomassen JC (2021). Reduced neutrophil elastase inhibitor elafin and elevated transforming growth factor-β_1_ are linked to inflammatory response in sputum of cystic fibrosis patients with Pseudomonas aeruginosa. ERJ Open Res.

[9] Lutful Kabir F (2018). MicroRNA-145 antagonism reverses TGF–β inhibition of F508del CFTR correction in airway epithelia. Am J Respir Crit Care Med.

[10] Snodgrass SM (2013). TGF–β1 inhibits Cftr biogenesis and prevents functional rescue of ΔF508-Cftr in primary differentiated human bronchial epithelial cells. PLoS One.

[11] Sun H (2014). TGF–β downregulation of distinct chloride channels in cystic fibrosis-affected epithelia. PLoS One.

[12] Fong CY (2000). TGF–β1 stimulates IL-8 release, COX2 expression, and PGE(2) release in human airway smooth muscle cells. Am J Physiol Lung Cell Mol Physiol.

[13] Park GY, Christman JW (2006). Involvement of cyclooxygenase-2 and prostaglandins in the molecular pathogenesis of inflammatory lung diseases. Am J Physiol Lung Cell Mol Physiol.

[14] Roca-Ferrer J (2006). Upregulation of COX1 and COX2 in nasal polyps in cystic fibrosis. Thorax.

[15] Lucidi V (2008). Exhaled 8-isoprostane and prostaglandin E(2) in patients with stable and unstable cystic fibrosis. Free Radic Biol Med.

[16] Konstan MW (1995). Effect of high-dose ibuprofen in patients with cystic fibrosis. N Engl J Med.

[17] Kim MD (2020). Losartan rescues inflammation-related mucociliary dysfunction in relevant models of cystic fibrosis. Am J Respir Crit Care Med.

[18] Manzanares D (2011). Functional apical large conductance, Ca2+-activated, and voltage-dependent K+ channels are required for maintenance of airway surface liquid volume. J Biol Chem.

[19] Manzanares D (2015). Airway surface dehydration by transforming growth factor β (TGF–β) in cystic fibrosis is due to decreased function of a voltage-dependent potassium channel and can be rescued by the drug pirfenidone. J Biol Chem.

[20] Bengtson CD (2021). Hyperglycaemia in CF adversely affects BK channel function critical for mucus clearance. Eur Respir J.

[21] McDougall CM (2008). Nasal epithelial cells as surrogates for bronchial epithelial cells in airway inflammation studies. Am J Respir Cell Mol Biol.

[22] Pranke IM (2017). Correction of CFTR function in nasal epithelial cells from cystic fibrosis patients predicts improvement of respiratory function by CFTR modulators. Sci Rep.

[23] McCague AF (2019). Correlating cystic fibrosis transmembrane conductance regulator function with clinical features to inform precision treatment of cystic fibrosis. Am J Respir Crit Care Med.

[24] Clancy JP (2019). CFTR modulator theratyping: current status, gaps and future directions. J Cyst Fibros.

[25] Megiorni F (2013). Elevated levels of miR-145 correlate with SMAD3 down-regulation in cystic fibrosis patients. J Cyst Fibros.

[26] Oglesby IK (2013). Regulation of cystic fibrosis transmembrane conductance regulator by microRNA-145, -223, and -494 is altered in ΔF508 cystic fibrosis airway epithelium. J Immunol.

[27] Futaki N (1994). NS-398, a new anti-inflammatory agent, selectively inhibits prostaglandin G/H synthase/cyclooxygenase (COX2) activity in vitro. Prostaglandins.

[28] Carlile GW (2015). Ibuprofen rescues mutant cystic fibrosis transmembrane conductance regulator trafficking. J Cyst Fibros.

