# Supplementary material for: Losartan ameliorates TGF-β1–induced CFTR dysfunction and improves correction by cystic fibrosis modulator therapies
Source: J Clin Invest. 2022 Jun 1;132(11):e155241. doi: 10.1172/JCI155241 (PMC9151698; doi:10.1172/JCI155241)
Supplement: Supplemental data [file jci-132-155241-s075.pdf]

Supplemental Material

Losartan ameliorates TGF-β1-induced CFTR dysfunction and improves correction by cystic fibrosis modulator therapies

Michael D. Kim[1,*], Charles D. Bengtson[1,*], Makoto Yoshida[1], Asef Niloy[1], John S. Dennis[1], Nathalie Baumlin[1], Matthias Salathe[1]

[1]Department of Internal Medicine, Division of Pulmonary, Critical Care and Sleep Medicine, University of Kansas Medical Center, Kansas City, KS 66160, USA.

[*]Contributed equally

Correspondence: Matthias Salathe, Department of Internal Medicine, University of Kansas Medical Center, 3901 Rainbow Blvd., 4032 Delp, MS 1022, Kansas City, KS 66160, USA.

Email: msalathe@kumc.edu.

Supplemental Methods

Lungs

Most CF bronchial epithelial (CFBE) cells were obtained from explanted lungs of appropriately consented CF patients undergoing lung transplantation, approved by the University of Miami Institutional Review Board. Lungs from one deceased CF donor were procured through LifeCenter Northwest (Bellevue, WA, USA). All normal human bronchial epithelial (NHBE) cells were obtained from non-smoking organ donors whose lungs were deemed unsuitable for transplantation but free of any known lung disease. Lungs were appropriately consented for donation by the organ procurement agencies, including LifeCenter Northwest, the Life Alliance Organ Recovery Agency at the University of Miami (Miami, FL, USA), the Nevada Donor Network (Las Vegas, NV, USA), and the Midwest Transplant Network (Westwood, KS, USA).

Air-liquid interface (ALI) cultures

Culturing of CFBE and NHBE cells at the ALI was performed as previously described by us and others with some modifications (1-3). CFBE cells were first expanded in PneumaCult™-Ex Plus Medium (STEMCELL Technologies, Cambridge, MA, USA). A second expansion was done in bronchial epithelial cell growth medium (BEGM) before passaging them on collagen-coated 12 mm Transwell inserts (#3460; Corning, Corning, NY, USA) or 12 mm Snapwell inserts (#3801; Corning) at a minimum of 150,000 cells/cm^2 in ALI media. Preparation of ALI media was adapted from published methods (1). CFBE cells were maintained submerged in ALI media for 4-6 days before exposing them to air. Cells were re-differentiated at an ALI for a minimum of three weeks before experiments were performed. For Ussing chamber experiments in Figures 1 and 2, both NHBE and CFBE cells were cultured as above but substituting ALI media with Vertex ALI media (4, 5) for the first eight days after plating on inserts. The apical surface of ALI cultures was washed with Dulbecco's phosphate-buffered saline (DPBS, #21-030-CV; Corning) and the basolateral media was changed three times per week.

Reagents

Elexacaftor (VX-445, #S8851), tezacaftor (VX-661, #S7059), ivacaftor (VX-770, #S1144), lumacaftor (VX-809; #S1565), and galunisertib (LY2157299; #S223) were acquired from Selleckchem (Houston, TX, USA). Recombinant human TGF-β1 protein (#240-B) and recombinant human TNF-α protein (#210-TA) were acquired from R&D Systems (Minneapolis, MN, USA). Losartan (losartan potassium; #61188) was acquired from MilliporeSigma (Burlington, MA, USA). NS-398 (#70590) and losartan carboxaldehyde (EXP3179; #18855) was acquired from Cayman Chemical (Ann Arbor, MI, USA).

Ussing chamber

Cystic fibrosis transmembrane conductance regulator (CFTR) and large conductance, Ca^{2+}-activated and voltage-dependent K^+ channel (BK) activities were recorded in Ussing chambers as previously described (6-8). For measurements of CFTR currents with elexacaftor/tezacaftor/ivacaftor (ETI) or lumacaftor/ivacaftor, ENaC currents were blocked by 10 µM amiloride (#A7410; MilliporeSigma) before addition of 1 µM VX-770 (#S1144; Selleckchem). CFTR current was stimulated by 10 µM forskolin (#F3197; MilliporeSigma) followed by inhibition with 10 µM $CFTR_{inh}$-172 (#C2992; MilliporeSigma). CaCC was stimulated by 100 µM UTP (ab146222; Abcam, Cambridge, MA, USA). For measurements of BK conductance with tezacaftor/ivacaftor or ETI, ENaC currents were blocked by 10 µM amiloride before addition of 1 µM VX-770 as above. BK current was stimulated by 10 µM ATP (#A9187; MilliporeSigma). The transepithelial membrane potential was clamped at 0 mV (model VCC MC8; Physiologic Instruments, San Diego, CA, USA) using Ag/AgCl electrodes in agar bridges. Signals were digitized and recorded using the Acquire and Analyze revision II module according to the manufacturer’s instructions.

ASL volume measurement

Meniscus scanning of high-resolution images of ALI cultures was used to estimate ASL volumes as previously described (9). The apical surface of ALI cultures was washed with DPBS and ASL volumes were measured after 24 hours. The basolateral media was replenished with media containing ETI + TGF-β1 ± losartan and ASL volumes were measured again after an additional 24 hours. ΔASL represents the difference between these two measurements with a more negative value indicating greater ASL absorption.

Mucus concentration (% solids) measurement

Mucus concentration (percent solids) was determined by published methods for measuring the wet and dry weights of mucus using a microbalance with accuracy to 100 ng (10, 11). Mucus was lifted off the cultures with a laser-cut mesh that was left on the surface of ALI cultures for 10 minutes at 37°C in an incubator.

Quantitative PCR

CFBE cells and nasal epithelial cells (see below) were lysed, and total RNA isolated using the E.Z.N.A.® Total RNA Kit (Omega Bio-tek, Norcross, GA, USA). Quantitative PCR (qPCR) was performed as described using TaqMan Gene Expression Assays (ThermoFisher Scientific, Waltham, MA, USA) for *CFTR* (Hs00357011_m1), *COX-2* (Hs00153133_m1), *IL1B* (Hs01555410_m1), *IL6* (Hs00985639_m1), *IL8* (Hs00174103_m1), *TGFBR1* (Hs00610320_m1), *TGFBR2* (Hs00234253_m1), and *TNFA* (Hs00174128_m1) and normalized to reference gene *GAPDH*. microRNA was isolated from CFBE cells using the miRNeasy Micro Kit (Qiagen, Germantown, MD, USA). *miR-145* expression was evaluated using TaqMan Advanced miRNA Assays (Assay ID 002278) and normalized to *RNU6B* (or *U6* snRNA).

ELISA

Basolateral media was collected from CFBE cells 24 hours after treatment. Media samples were analyzed using the Ella Automated Immunoassay System and Simplex Plex cartridge-based immunoassay for TNFα (Bio-Techne Corp., Minneapolis, MN, USA). Activated and total TGF-β1 protein levels were measured from nasal epithelial lining fluid (see below) with a Simplex Plex cartridge-based immunoassay for TGF-β1 (Bio-Techne Corp.). Total TGF-β1 was measured by activating latent TGF-β1 with acidification and neutralization steps before running the assay. Levels of active TGF-β1 are expressed as a ratio of active/total TGF-β1.

Study approval and enrollment criteria

The study protocol was approved by the University of Kansas Medical Center Institutional Review Board and informed consent was obtained from each participant.

Participants and Study Procedures

Those with CF age 18 years and older at the University of Kansas Health System on a stable dose of ETI for at least three months were enrolled. Participants were excluded if they had undergone treatment for a CF pulmonary exacerbation in the past month, were currently taking systemic corticosteroids or other anti-inflammatory medications, or had recent nose bleeding. Demographics and clinical parameters were obtained from the electronic medical record. Lung function response to ETI was determined by the change, both relative and absolute, in the percent predicted forced expiratory volume in one second (ppFEV1) between the average of the three highest ppFEV1 measurements in the year preceding and year following initiation of ETI. Sweat was obtained through pilocarpine disc iontophoresis (Macroduct®, ELITechGroup, Logan, UT, USA). Sweat chloride levels were measured in a CLIA certified laboratory.

Nasal fluid collection (Leukosorb)

Collection of nasal epithelial lining fluid (ELF) was performed with pre-cut strips of synthetic absorptive filter (Leukosorb; Pall Corporation, Port Washington, NY, USA). Leukosorb strips were inserted under direct visual guidance into the nasal cavity of CF participants with the entire length of the strip applied laterally against the anterior inferior turbinate. Nose clips were used to ensure good contact with the mucosal surface for two minutes. After removal from the nose, Leukosorb strips were placed in an Eppendorf tube and stored at -80ºC before elution as previously described (12).

Nasal cells collection

Nasal cells were collected using sterile cytology brushes (Medical Packaging Corporation, Camarillo, CA, USA). The brushes were introduced under direct visual guidance into the nasal cavity of CF participants and were placed between the nasal septum and the inferior turbinate. No anesthesia was used. The cells were harvested by a few careful backward-forward and rotary movements before twirling the brush into 5 mL of sterile PBS in a 15 mL tube to release the cells. The same procedure was repeated 3 times in each nostril. Immediately after the harvest, the tube was centrifuged at 360 x g for 5 min at 4°C. The supernatant was discarded, and the remaining pellet was frozen at -80°C until qPCR experiments were performed.

Statistical analyses

Statistics: Demographics and baseline clinical characteristics were summarized with descriptive statistics. Pearson's or Spearman's correlation coefficient with one-tailed or two-tailed p values (see figure legends), depending on the distribution of the data was used to assess correlation between continuous variables. Two-tailed Student's t-test and one way ANOVA were

used otherwise. All results were considered statistically significant if $p < 0.05$. All analysis was conducted using Prism (GraphPad Software, San Diego, CA, USA).

Study approval. The study protocol was approved by the University of Kansas Medical Center Institutional Review Board and informed consent was obtained from each participant.

Supplemental Table S1. CF lung donor information.

Sex	Age	CFTR Mutation
Female	17	F508del/F508del
Female	36	F508del/F508del
Male	18	F508del/G551D
Male	27	F508del/F508del
Male	30	F508del/F508del
Male	38	F508del/F508del
Male	45	F508del/F508del
Male	54	F508del/F508del

Supplemental Figure S1.

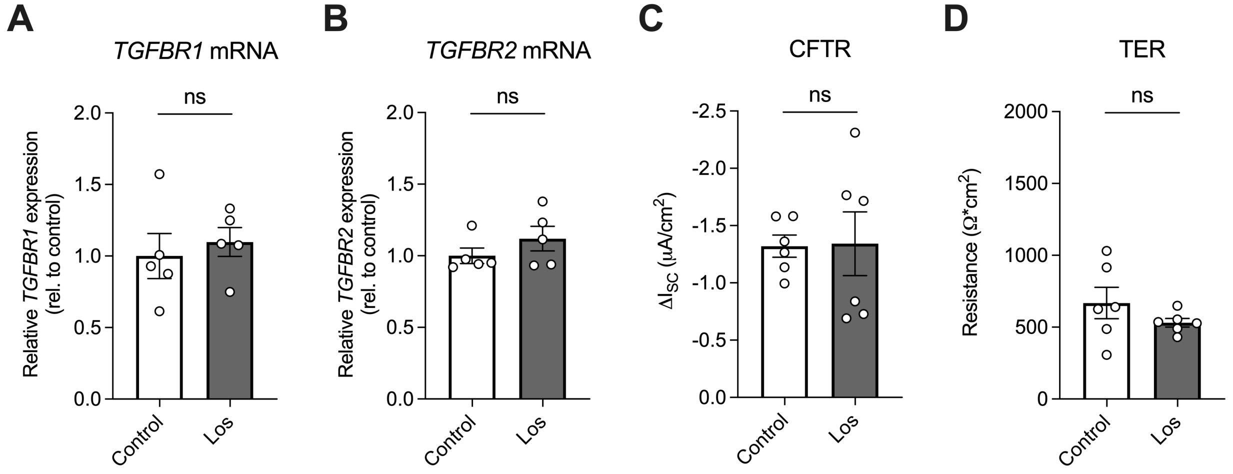

Supplemental Figure S1. Effects of chronic losartan treatment on *TGFBR1* and *TGFBR2* mRNA expression, CFTR conductance, and transepithelial resistance (TER) in homozygous F508del CFBE cells *in vitro*. (**A, B**) Chronic basolateral losartan (10 µM) does not change mRNA expression levels of *transforming growth factor beta receptor 1/2* (*TGFBR1/2*) in fully differentiated homozygous F508del CFBE cells. n=5 CF lungs. (**C,D**) Chronic basolateral losartan does not change F508del-CFTR conductance (**C**) or transepithelial resistance (TER) (**D**) in fully differentiated homozygous F508del CFBE cells. n=6, 2 CF lungs. *Statistics*: Data are shown as mean ± SEM. * $p<0.05$, ns=not significant. Student's t-test after assessing normality by Shapiro-Wilk.

Supplemental Figure S2.

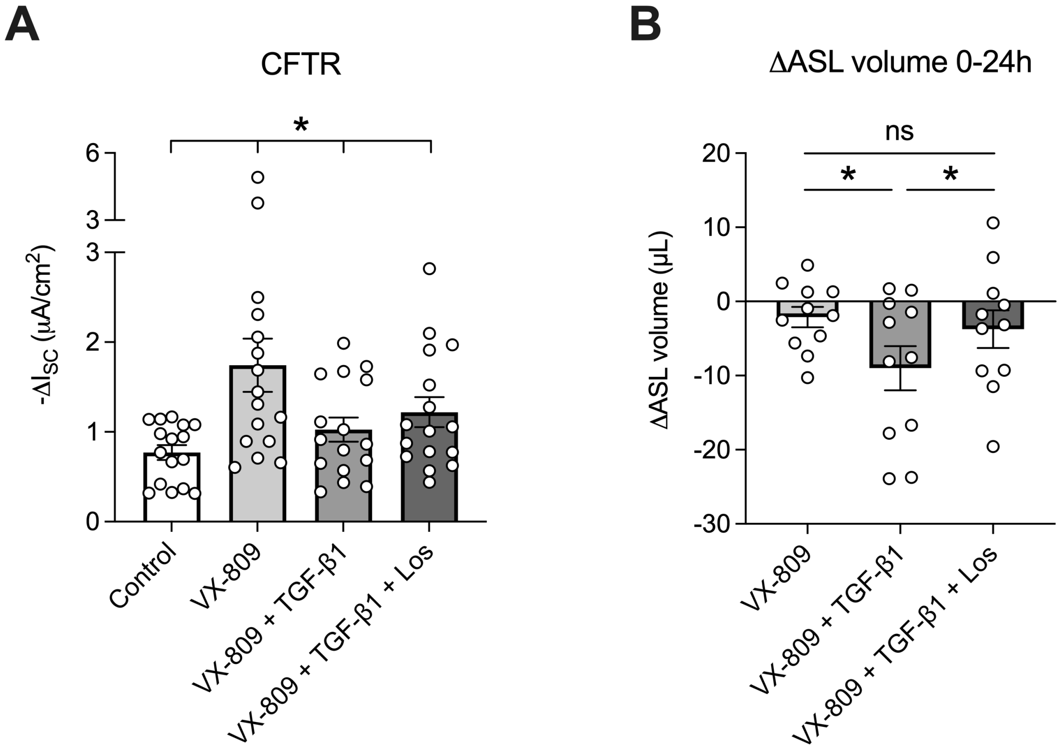

Supplemental Figure S2. Losartan partially rescues TGF-β1 inhibition of lumacaftor (VX-809)-corrected F508del-CFTR function and TGF-β1-induced ASL absorption in homozygous F508del CFBE cells *in vitro*. (**A**) Basolateral TGF-β1 (10 ng/mL) causes a significant decrease in lumacaftor (VX-809)-corrected F508del-CFTR conductance that is partially rescued by losartan (10 μM). n=16, 7 CF lungs. (**B**) Basolateral TGF-β1 induces greater ASL absorption (as indicated by a more negative ΔASL volume) in lumacaftor-treated CFBE cells after 24 hours. The TGF-β1-induced increase in ASL absorption is reversed by losartan. n=11, 6 CF lungs. *Statistics*: Data are shown as mean ± SEM. * $p<0.05$, ns=not significant. All groups are significantly different from each other in **A**. One-way ANOVA followed by Holm-Sidak after assessing normality by Shapiro-Wilk.

Supplemental Figure S3.

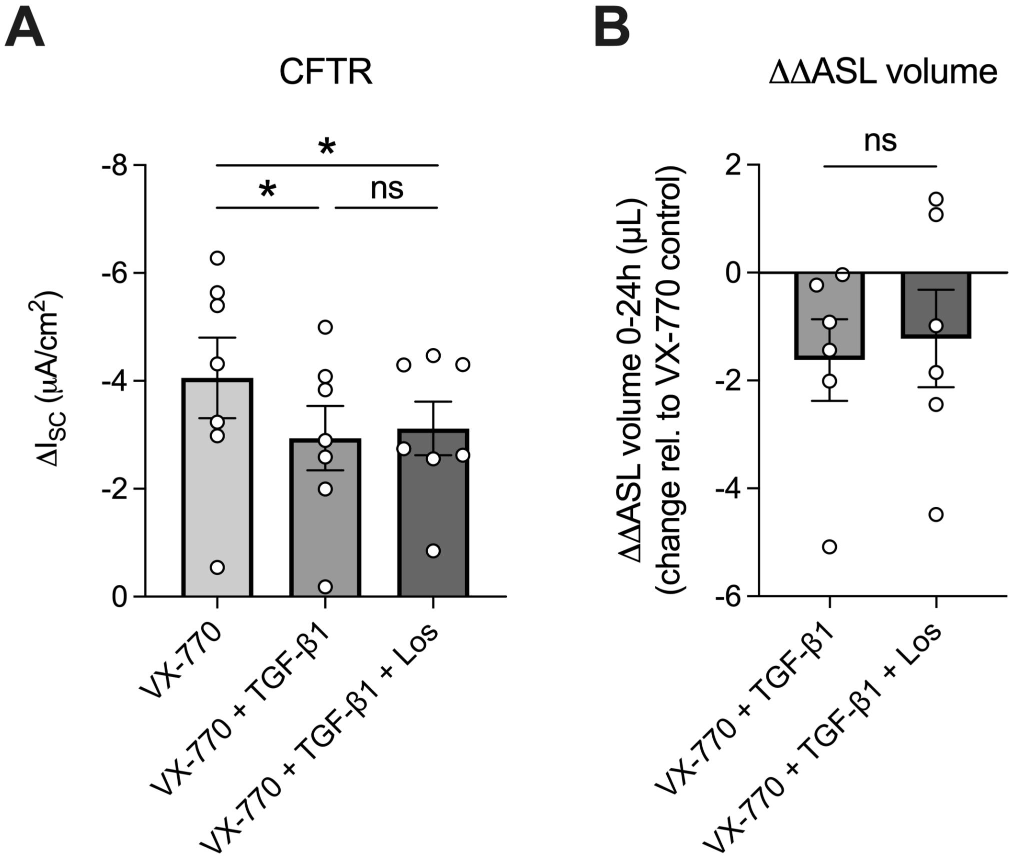

Supplemental Figure S3. Losartan does not rescue TGF-β1 inhibition of ivacaftor (VX-770)-potentiated G551D-CFTR function and TGF-β1-induced ASL absorption in G551D/F508del CFBE cells *in vitro*. (**A**) Basolateral TGF-β1 (5 ng/mL) causes a significant decrease in VX-770-potentiated G551D-CFTR conductance that is not rescued by chronic losartan (10 µM) in G551D/F50del CFBE cells. n=7, 1 CF lung. (**B**) Change in ASL volume (ΔASL volume) depicted as change in volume relative to VX-770 control (ΔΔASL volume). Chronic losartan does not improve ASL absorption induced by TGF-β1 in VX-770-treated G551D/F508del CFBE cells after 24 hours. n=6, 1 CF lung. *Statistics*: Data are shown as mean ± SEM. * $p<0.05$, ns=not significant. One-way ANOVA followed by Holm-Sidak after assessing normality by Shapiro-Wilk.

Supplemental Figure S4.

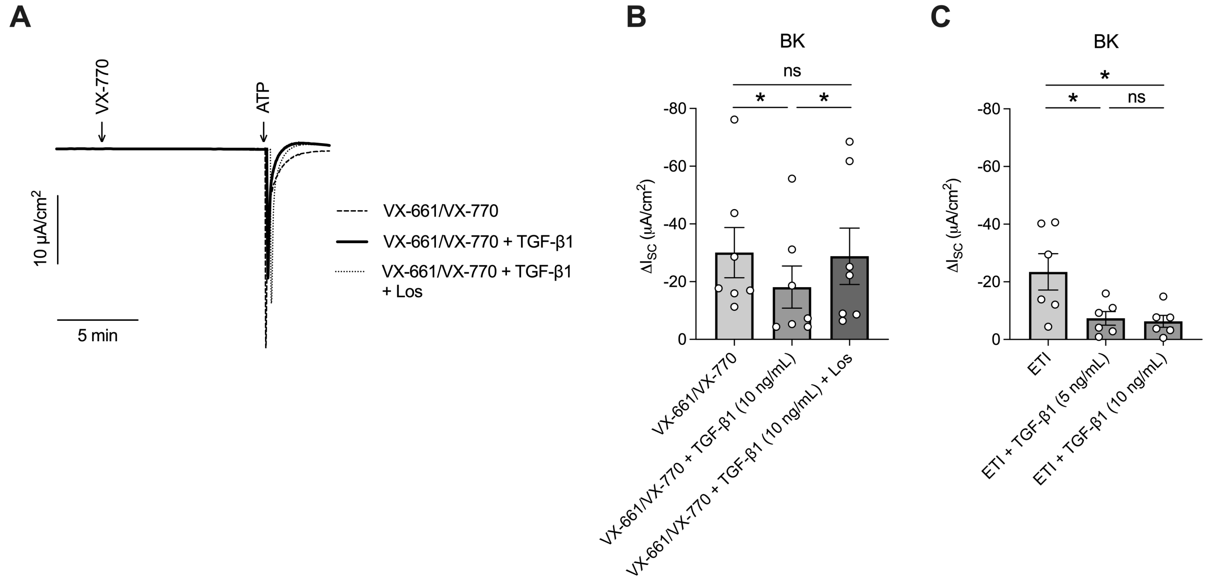

Supplemental Figure S4. Losartan partially rescues BK channel function in TGF-β1- and tezacaftor/ivacaftor-treated homozygous F508del CFBE cells *in vitro*. (**A**) Representative tracing of ATP-stimulated I_{SC} with a basolateral-to-apical K^+ and apical to basolateral Na^+ gradient from fully differentiated homozygous F508del CFBE cells treated with tezacaftor (VX-661; 5 μM)/ivacaftor (VX-770; 1μM), VX-661/VX-770 + TGF-β1 (10 ng/mL), or VX-661/VX-770 + TGF-β1 + losartan (10 μM). (**B**) Losartan (Los) rescues TGF--β1-induced decreases in BK channel function in VX-661/VX-770-treated homozygous F508del CFBE cells. CFBE cells were treated with basolateral losartan for ≥ 3 weeks. n=7, 4 CF lungs. (**C**) Basolateral TGF-β1 causes a significant reduction in BK channel conductance at concentrations of 5 ng/mL and 10 ng/mL in ETI-treated F508del CFBE cells after 24 hours. *Statistics*: Data are shown as mean ± SEM. * $p<0.05$, ns=not significant. Friedman test for **B** and one-way ANOVA followed by Holm-Sidak for **C** after assessing normality by Shapiro-Wilk.

Supplemental Figure S5.

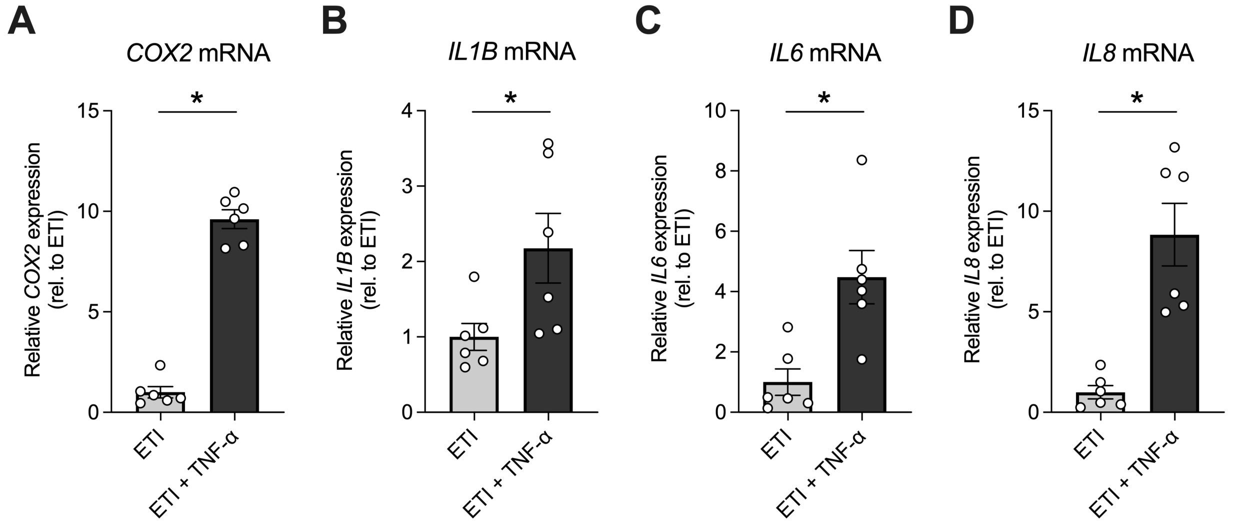

Supplemental Figure S5. TNF-α induces a significant increase in the mRNA expressions of inflammatory markers. Basolateral TNF-α (10 ng/mL) induces a significant increase in expression levels of *COX2* (**A**), *IL1B* (**B**), *IL6* (**C**), and *IL8* (**D**) mRNAs in ETI-treated F508del CFBE cells after 24 hours. n=6, 3 CF lungs. *Statistics*: Data are shown as mean ± SEM. * $p < 0.05$. Wilcoxon test for **A** and Student's t-test for **B-D** after assessing normality by Shapiro-Wilk.

Supplemental Figure S6.

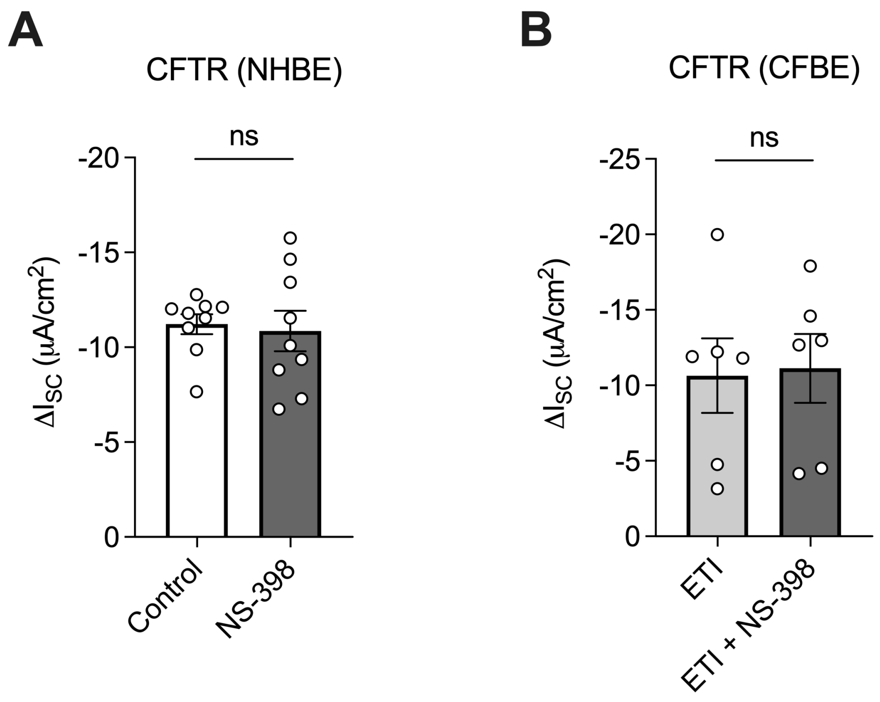

Supplemental Figure S6. Effects of the COX-2 inhibitor NS-398 on wild-type CFTR and ETI-corrected F508del-CFTR function. (**A**) 24-hour treatment with NS-398 (10 µM) does not significantly change CFTR conductance in fully differentiated normal human bronchial epithelial (NHBE) cells cultured at the ALI. n=9, 3 normal lungs. (**B**) ETI-corrected F508del-CFTR function is statistically unchanged after 24-hour treatment with NS-398 (10 µM) in fully differentiated homozygous F508del CFBE cells. n=6, 5 CF lungs. *Statistics*: Data are shown as mean ± SEM. ns=not significant. Wilcoxon test for **A** and student's t-test for **B** after assessing normality by Shapiro-Wilk.

References

1. Randell SH, Fulcher ML, O'Neal W, and Olsen JC. Primary epithelial cell models for cystic fibrosis research. *Methods Mol Biol.* 2011;742:285-310.
2. Sutto Z, Conner GE, and Salathe M. Regulation of human airway ciliary beat frequency by intracellular pH. *J Physiol.* 2004;560(Pt 2):519-32.
3. Schmid A, Sutto Z, Schmid N, Novak L, Ivonnet P, Horvath G, et al. Decreased soluble adenylyl cyclase activity in cystic fibrosis is related to defective apical bicarbonate exchange and affects ciliary beat frequency regulation. *J Biol Chem.* 2010;285(39):29998-30007.
4. Neuberger T, Burton B, Clark H, and Van Goor F. Use of primary cultures of human bronchial epithelial cells isolated from cystic fibrosis patients for the pre-clinical testing of CFTR modulators. *Methods Mol Biol.* 2011;741:39-54.
5. Gentzsch M, Boyles SE, Cheluvaraju C, Chaudhry IG, Quinney NL, Cho C, et al. Pharmacological Rescue of Conditionally Reprogrammed Cystic Fibrosis Bronchial Epithelial Cells. *Am J Respir Cell Mol Biol.* 2017;56(5):568-74.
6. Bengtson CD, Kim MD, Anabtawi A, He J, Dennis JS, Miller S, et al. Hyperglycaemia in CF adversely affects BK channel function critical for mucus clearance. *Eur Respir J.* 2020.
7. Manzanares D, Gonzalez C, Ivonnet P, Chen RS, Valencia-Gattas M, Conner GE, et al. Functional apical large conductance, Ca2+-activated, and voltage-dependent K+ channels are required for maintenance of airway surface liquid volume. *J Biol Chem.* 2011;286(22):19830-9.
8. Manzanares D, Krick S, Baumlin N, Dennis JS, Tyrrell J, Tarran R, et al. Airway Surface Dehydration by Transforming Growth Factor beta (TGF-beta) in Cystic Fibrosis Is Due to Decreased Function of a Voltage-dependent Potassium Channel and Can Be Rescued by the Drug Pirfenidone. *J Biol Chem.* 2015;290(42):25710-6.
9. Harvey PR, Tarran R, Garoff S, and Myerburg MM. Measurement of the airway surface liquid volume with simple light refraction microscopy. *Am J Respir Cell Mol Biol.* 2011;45(3):592-9.
10. Anderson WH, Coakley RD, Button B, Henderson AG, Zeman KL, Alexis NE, et al. The Relationship of Mucus Concentration (Hydration) to Mucus Osmotic Pressure and Transport in Chronic Bronchitis. *Am J Respir Crit Care Med.* 2015;192(2):182-90.
11. Kim MD, Baumlin N, Yoshida M, Polineni D, Salathe SF, David JK, et al. Losartan Rescues Inflammation-related Mucociliary Dysfunction in Relevant Models of Cystic Fibrosis. *Am J Respir Crit Care Med.* 2020;201(3):313-24.
12. Rebuli ME, Speen AM, Clapp PW, and Jaspers I. Novel applications for a noninvasive sampling method of the nasal mucosa. *Am J Physiol Lung Cell Mol Physiol.* 2017;312(2):L288-L96.
